# *Pearson*’s patterns correlational of clinical risks at admissions with hospitalization outcomes during initial COVID-19 outbreak

**DOI:** 10.1016/j.isci.2022.104415

**Published:** 2022-05-18

**Authors:** Jingwen Li, Xi Long, Qing Zhang, Xi Fang, Huiling Luo, Fang Fang, Xuefei Lv, Dandan Zhang, Yu Sun, Na Li, Shaoping Hu, Jinghong Li, Nian Xiong, Zhicheng Lin

**Affiliations:** 1 Union Hospital, Tongji Medical College, Huazhong University of Science and Technology, Wuhan, Hubei, China; 2 Wuhan Red Cross Hospital, Wuhan, Hubei, China; 3 Department of Radiology, Union Hospital, Tongji Medical College, Huazhong University of Science and Technology, Wuhan, Hubei, China; 4 Department of Anesthesiology, Wuhan Red Cross Hospital, Wuhan, Hubei, China; 5 Department of Radiology, Wuhan Red Cross Hospital, Wuhan, Hubei, China; 6 Department of Medicine, University of California San Diego, La Jolla, CA 92093, USA; 7 McLean Hospital, Harvard Medical School, Belmont, MA 02478, USA

**Keywords:** Health sciences, Clinical finding, Risk factor

## Abstract

COVID-19 outbreaks have crushed our healthcare systems, which requires clinical guidance for the healthcare following the outbreaks. We conducted retrospective cohort studies with *Pearson*’s pattern-based analysis of clinical parameters of 248 hospitalized patients with COVID-19. We found that dysregulated neutrophil densities were correlated with hospitalization duration before death (p = 0.000066, *r* = −0.45 for % neutrophil; p = 0.0001, *r* = −0.47 for neutrophil count). As such, high neutrophil densities were associated with mortality (p = 4.23 × 10^−31^ for % neutrophil; p = 4.14 × 10^−27^ for neutrophil count). These findings were further illustrated by a representative “second week crash” pattern and validated by an independent cohort (p = 5.98 × 10^−11^ for % neutrophil; p = 1.65 × 10^−7^ for neutrophil count). By contrast, low aspartate aminotransferase (AST) or lactate dehydrogenase (LDH) levels were correlated with quick recovery (p ≤ 0.00005). Collectively, these correlational at-admission findings may provide healthcare guidance for patients with COVID-19 in the absence of targeted therapy.

## Highlights

• Neutrophile surge alone can be a significant risk for COVID-19 mortality
• High AST, LDH, or ALT level at admission predicts longer hospitalization
• Inhibition of neutrophil represents a therapeutical mechanism

## Graphical abstract

**Figure:**
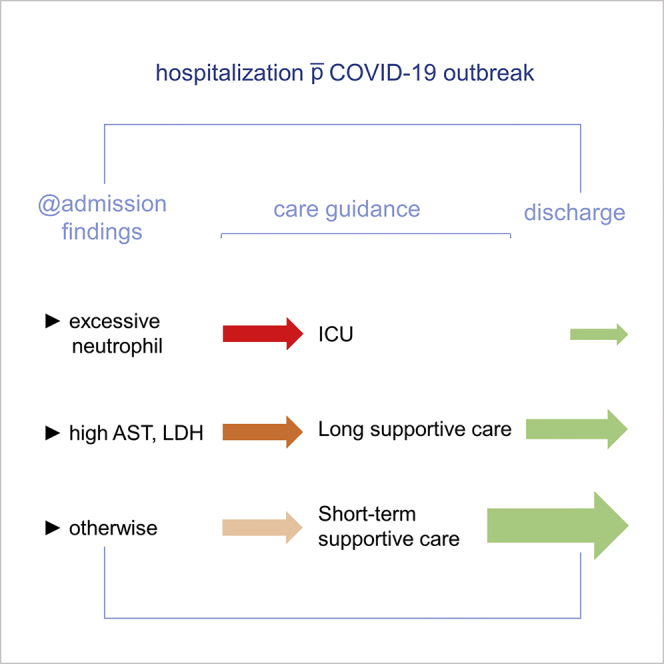

## Introduction

The widespread COVID-19 has caused hundred million infections, more than 2% of them dead, and crushed our healthcare capacity in many regions (Patrucco et al., 2021; Rubin, 2020). This pandemic has demonstrated the need to identify clinical risk factors that could foresee hospital outcomes for patients with novel infectious diseases such as COVID-19. This became particularly true in the absence of effective therapy, depending only on supportive care, oxygen therapy, noninvasive mechanical ventilation, and invasive mechanical ventilation (Alhumaid et al., 2020).

Many studies have identified clinical characteristics as risk factors associated with severe COVID-19, which include aging, male gender, comorbidities, high D-dimer, C-reactive protein (CRP), lactate dehydrogenase (LDH), and white blood cells (WBCs) levels and low lymphocyte levels (Ou et al., 2020; Parohan et al., 2020). No comprehensive patterns/correlational study has been carried out.

**Figure 1:**
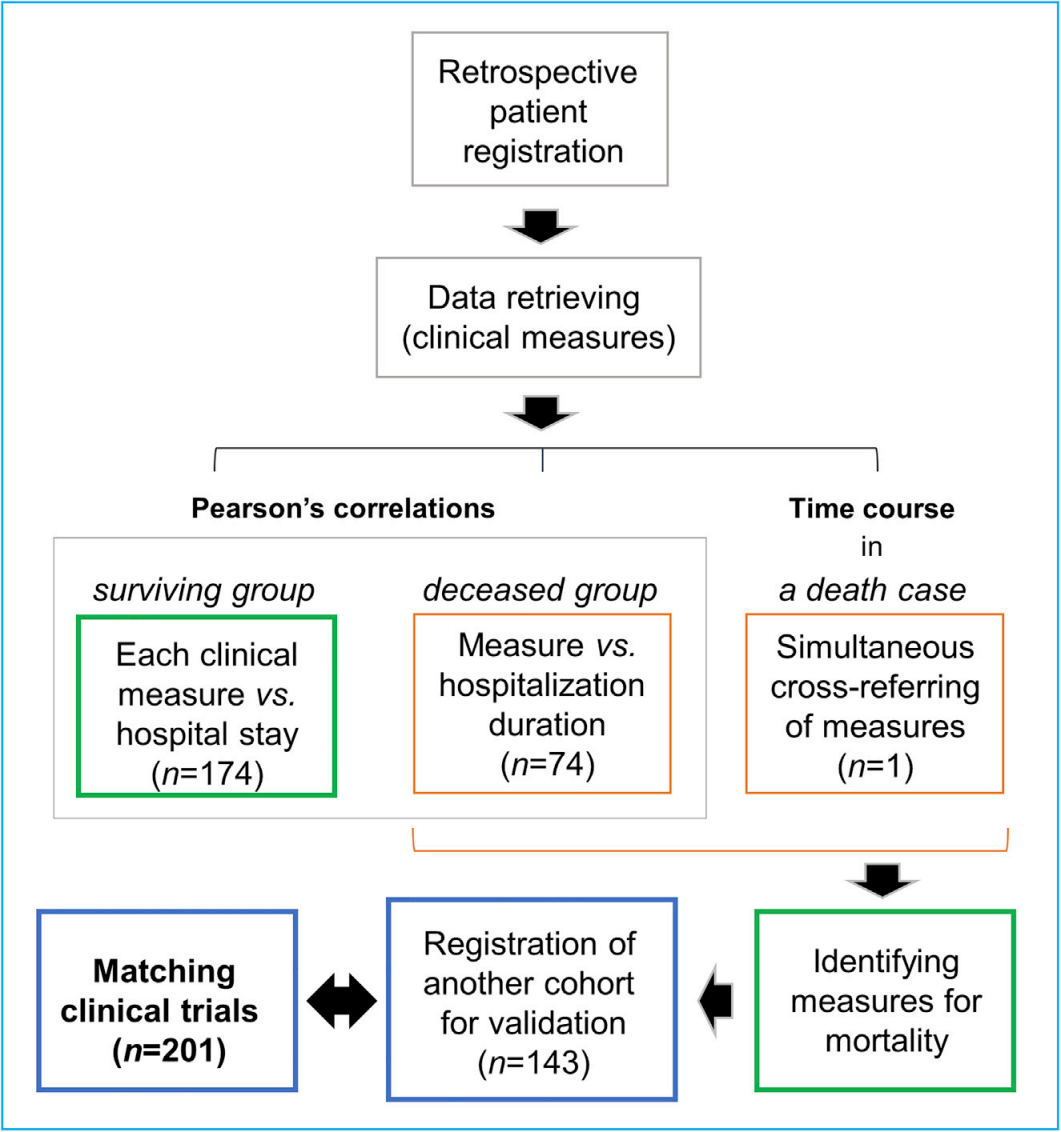
Design for dissecting pathogenicity factors (green), followed by correlational and clinical trial validations (blue)

The purpose of this retrospective cohort study, as outlined in Figure 1, was to, by *Pearson*’s correlation, comprehensively delineate the prognostic markers for hospitalization duration and mortality by correlating disease history, vital signs, and laboratory (27 *in toto*) parameters for initial COVID-19 patients when both healthcare workers and patients were in panic. We studied two independent cohorts, each with a surviving group versus a deceased group of the COVID-19 patients. We found laboratory results from the blood could serve as prognostic markers for hospital outcomes. We also did an extensive database search of ongoing clinical trials for marker-related therapies.

## Results

### Clinical characteristics of study cohort at admission

The retrospective cohort study has two groups: 174 patients survived and were discharged from hospital as the surviving group and 74 patients died as the deceased group. All patients were confirmed with COVID-19 and were hospitalized at Wuhan Red Cross Hospital (WRCH) in Wuhan, China. Most of the examined clinical characteristics including age, gender, signs and symptoms, and laboratory results at admission are shown in Table 1a. The median age of the deceased group was 20 years older than the surviving group. Among 74 deceased patients, 81% were over 60 years old and males accounted for the majority (65%). The two groups had similar symptoms of fever and cough at admission, which were the most common symptoms among all the patients. For the deceased group, they had significantly higher rate of shortness of breath, fatigue, and myalgia. For the surviving group, they had more gastrointestinal tract symptoms such as nausea, vomiting, and diarrhea.

**Table 1:** At-admission characteristics of survivors versus deceased cases: clinical manifestations (a) and laboratory results (b)

(a) Main demographics and clinical manifestations^a^				
NO. (%)	Total patients (n = 248)	Surviving cases (n = 174)	Deceased cases (n = 74)	p value
Age, years (median, range)	**54.6**	47.5 (22, 87)	67.5 (31, 87)	***<0.0001***

**Gender**				

Female	126	100 (57%)	26 (35%)	***<0.0001***
Male	122	74 (43%)	48 (65%)	***0.0001***

**Signs and symptoms at admission**				

Fever	193	134 (77%)	59 (80%)	ns
Cough	165	123 (71%)	42 (57%)	***0.0334***
Shortness of breath	129	57 (33%)	72 (97%)	***<0.0001***
Fatigue	136	76 (44%)	60 (81%)	***<0.0001***
Myalgia	41	32 (18%)	9 (12%)	***0.0045***
Headache	16	12 (7%)	4 (5%)	***0.0219***
Sore throat	23	19 (11%)	4 (5%)	ns
Chest pain	52	33 (19%)	19 (26%)	ns
Diarrhea	39	29 (17%)	10 (14%)	***0.0278***
Abdominal discomfort	14	11 (6%)	3 (4%)	***<0.0001***
Nausea and vomiting	13	10 (6%)	3 (4%)	***0.0042***
Anorexia	71	57 (33%)	14 (19%)	***<0.0001***
Dizziness	36	26 (15%)	10 (14%)	ns
More than one sign or symptom	215	143 (82%)	72 (97%)	***<0.0001***

(b) Common laboratory results^b^				
NO. (%)	Normal range	Surviving cases (n = 174)	Deceased cases (n = 74)	p value
**Blood routine**				

White blood cells (WBCs) count, ×109/L	3.5–9.5	4.64 (1.67, 11.78)	8.45 (2.34, 21.12)	***<0.0001***
Neutrophil percentage, %	50–70	60.5 (3.13, 94.7)	89.9 (47.5, 97.9)	***<0.0001***
Lymphocyte percentage, %	20–40	28.4 (2, 54)	6.25 (0.6, 42.3)	***<0.0001***
Platelet count, ×109/L	100–300	188 (55, 483)	149 (23, 448)	***0.0011***
Hemoglobin, g/L	110–150 (F); 120–160 (M)	135 (74, 314)	127.5 (46, 169)	***0.0044***

**Coagulation function**				

Prothrombin time (PT), s	9–13	12.3 (1.04, 28.2)	13.5 (10.2, 23)	***0.0002***
Activated partial thromboplastin time (APTT)	20–40	28.05 (1.03, 54.2)	32.35 (17.9, 66.8)	***<0.0001***
D-dimer, mg/L	<0.55	0.36 (0.1, 48.4)	4.43 (0.15, 170)	***<0.0001***

**Blood biochemistry**				

Blood urea nitrogen (BUN), mmol/L	3.1–8	3.6 (1.2, 9.8)	6.6 (2.2, 49)	***<0.0001***
Creatinine, μmmol/L	57–97	64.1 (33.1, 134.3)	73.1 (36.7, 1393.6)	***<0.0001***
Creatine kinase, U/L	24–185	54.9 (3.5, 1533.9)	111.3 (5.61, 2840.5)	***<0.0001***
Lactate dehydrogenase (LDH), U/L	90–245	182.75 (15.1, 922.2)	398.9 (63.9, 2309.8)	***<0.0001***
Alanine aminotransferase (ALT), U/L	0–40	20.8 (3.7, 163.8)	29.1 (4.6, 371)	***<0.0001***
Aspartate aminotransferase (AST), U/L	0–45	25 (6.3, 140.7)	44.9 (1.6, 236.9)	***<0.0001***
Total bilirubin, mmol/L	2–25	8.61 (1.14, 75.69)	12.43 (1.4, 71.8)	***0.0001***
Glucose, mmol/L	3.9–6.1	5.26 (4.11, 17.76)	7.89 (3.01, 24.21)	***<0.0001***

**Infection-related biomarkers**				

C-reactive protein (CRP), mg/L	<10	10.8 (0.1, 310.01)	81.2 (8.4, 284.4)	***<0.0001***
Erythrocyte sedimentation rate (ESR), mm/h	0–20 (F); 0–15 (M)	32 (1, 76)	44 (16, 97)	***0.0358***

Other measures analyzed included mean arterial pressure, heart rate, fever, breath rate, and neutrophil-to-lymphocyte ratio (NLR).

a Data are median, n (range), or *n* (%). p values comparing deceased patients and surviving patients were from χ^2^ tests or Fisher’s exact tests. p < 0.05 was considered statistically significant (in bold); ns, not significant.

b Data are median, n (range), or *n* (%). p values were from χ^2^ tests or Fisher’s exact tests. p < 0.05 was considered statistically significant (in bold).

Numerous laboratory findings were found significantly different between the surviving group and the deceased group (Table 1b). The deceased group had higher levels in several parameters, including elevated D-dimer (12.3-fold), CRP (7.5-fold), and LDH (2.2-fold). The deceased group had elevated total WBC count (1.8-fold), elevated neutrophil percentage (1.49-fold), and decreased lymphocyte percentages (0.2-fold). All of the above parameters were statistically significant (p < 0.0001) between the surviving and deceased groups. The abnormalities of these parameters are typically seen in severe COVID-19 patients from previous studies (Huang et al., 2020; Wu et al., 2020; Zhou et al., 2020). Therefore, the laboratory parameters confirmed that indeed the surviving group patients had mild disease and the deceased group patients had severe disease, respectively.

### Clinical characteristics correlated with hospitalization duration

A total of 32 clinical parameters were retrieved from the medical records (Table 1). Indexing powers for three aspects including surviving versus deceased, hospitalization duration before discharge for the survivors, and hospitalization duration before death for the deceased were examined.

**Figure 2:**
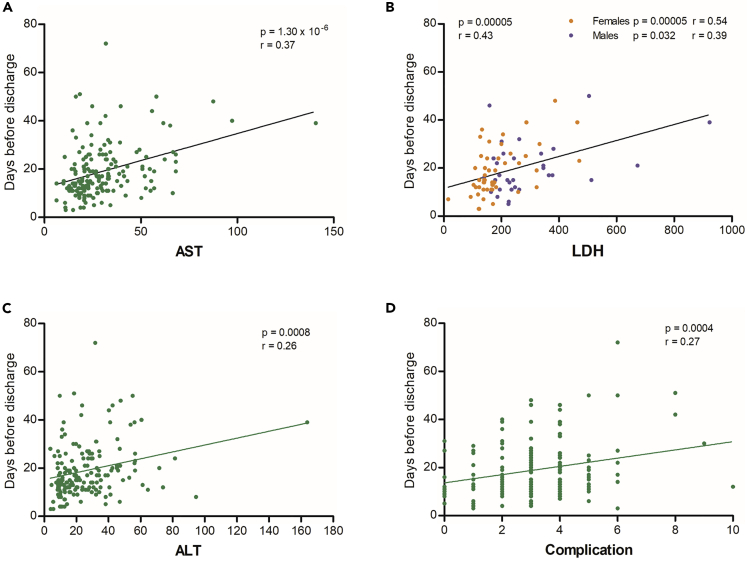
Concentration-dependent correlation of four measures with hospital days before discharge in the surviving group Each dot represents a patient. (A) AST (n = 168; best-fit slope mean 0.225 ± 0.045, 95%CI 0.137–0.312). (B) LDH (n = 81, 31 males and 50 females; gender combined slope mean 0.0333 ± 0.0076, 95%CI 0.0178–0.0487; female slope mean 0.0603 ± 0.0135, 95%CI 0.0330–0.0875; male slope mean 0.0250 ± 0.0011, 95%CI 0.00227–0.04779). (C) ALT (n = 167, slope mean 0.143 ± 0.042, 95%CI 0.0608–0.2250) and (D) complication (n = 170; slope mean 1.715 ± 0.476, 95%CI 0.782–2.648). All of them passed multiple-testing, but none of them survived multiple-testing for the deceased group.

For the surviving group, 4 of the 32 parameters displayed concentration- or abundance-dependent and positive associations with hospital days before discharge. The 4 parameters are aspartate aminotransferase (AST), LDH, ALT, and more than one sign or symptom (Figure 2). Furthermore, LDH displayed gender specificity: the correlation was much stronger in females (p = 0.00005) than males (p = 0.032, which failed multiple-testing). LDH was not a covariant of the other three parameters (p < 0.0001) but the other three were covariant with each other. The remaining 28 parameters were not covariant with hospital days before discharge. Other parameters such as age, D-dimer, neutrophil percentage, and lymphocyte count had correlational tendencies but they were not significant (data not shown).

**Figure 3:**
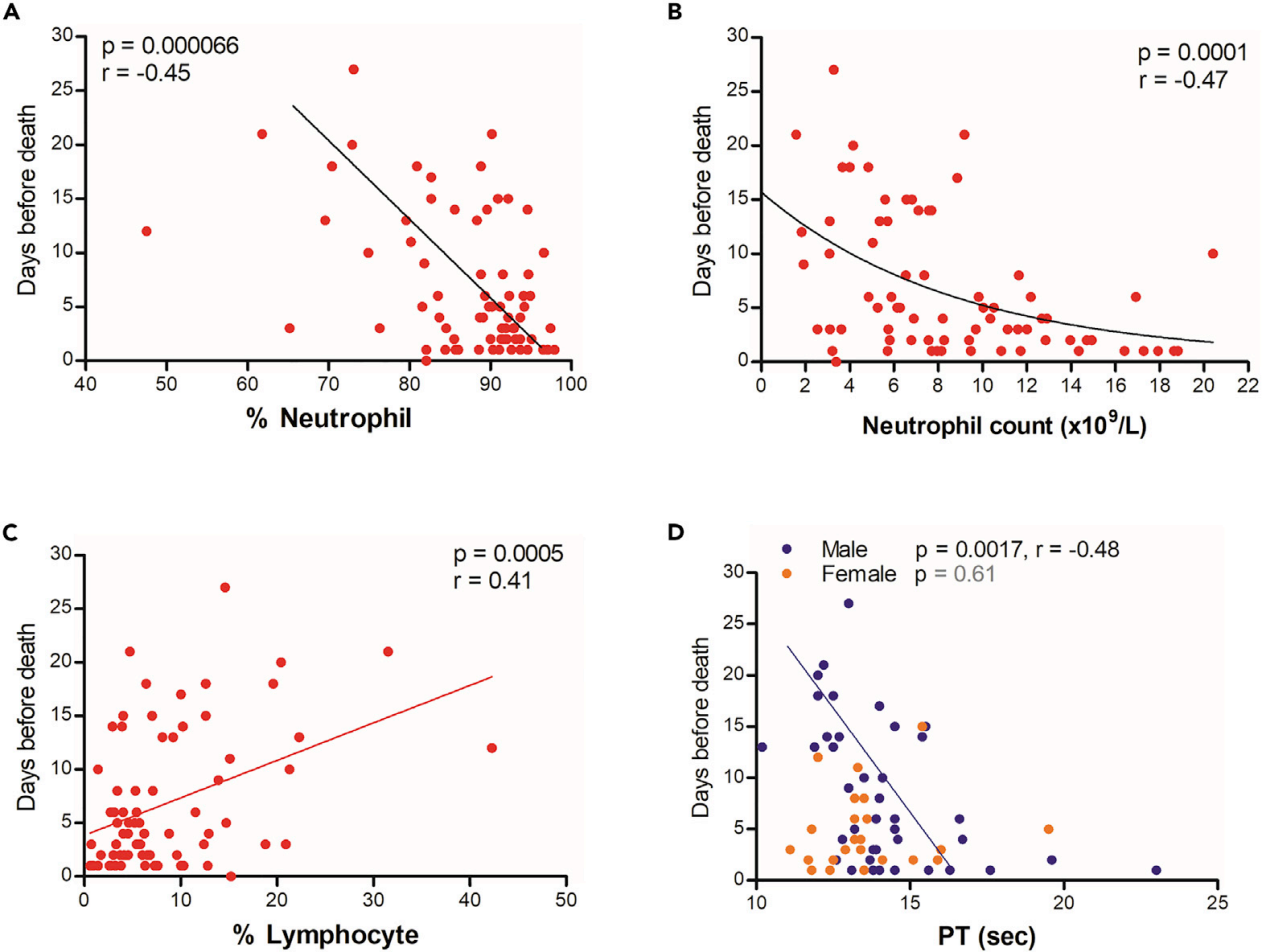
Concentration-dependent correlation of top measures with hospitalization duration before death in the deceased group Each dot represents a patient. (A) % neutrophil (n = 72; best-fit slope mean −0.321 ± 0.076, 95%CI −0.472∼−0.170). (B) Neutrophil count (n = 72; best-fit slope mean −0.606 ± 0.150, 95%CI −0.905∼−0.306; nonlinear Y0 = 15.69 with 95%CI 7.32 ± 24.07 and k = 0.114 with 95%CI 0.0–0.324). (C) % lymphocyte (n = 72; slope mean 0.350 ± 0.096, 95%CI 0.158–0.542) and (D) PT (n = 41 males and 25 females; male slope mean −1.489 ± 0.441, 95%CI −2.38∼−0.60) where p = 0.018, *r* = −0.29 after gender mixed. WBCs displayed a correlation similar to neutrophil count (p = 0.0002, *r* = −0.43, n = 72; slope mean −0.581 ± 0.150, 95%CI −0.880∼−0.281). None of them passed multiple-testing for the surviving group.

For the deceased group, 5 of the 32 parameters were concentration-dependently correlated with hospitalization duration before death. The top three specific parameters were neutrophil count, neutrophil percentage, and lymphocyte percentage (all p ≤ 0.0005) (Figures 3A–3C). The fifth one was prothrombin time (PT), which showed a significant correlation only in males (p = 0.0017) (Figure 3D). Lymphocyte percentage had positive correlation, and all others had negative correlations, as shown in Figure 3. These appeared to be covariant based on similar slopes. Therefore, all of these correlations reached statistical significances. The subset types of WBC, neutrophil count, neutrophil percentage, and lymphocyte percentage were correlated with the hospitalization duration before death. We have also examined neutrophil-to-lymphocyte ratio (NLR) and found no significant correlation (data not shown).

### Representative case illustration for time course of WBC subset type abundance

One of the clinical features we observed from COVID-19 patients was a portion of patients presented with mild disease; they could be feeling better and then *suddenly* getting *worse* around two weeks into the disease course. This is so called “second week crash.” It was thought that these patients might have suffered from cytokine storm (Fajgenbaum and June, 2020). We analyzed a case like this to study the time course. Besides concentration-dependence, time course may allow simultaneous cross-referring among multiple parameters. This patient presented with mild severity at admission but worsened within 2–3 weeks, leading to death within 26 days.

**Figure 4:**
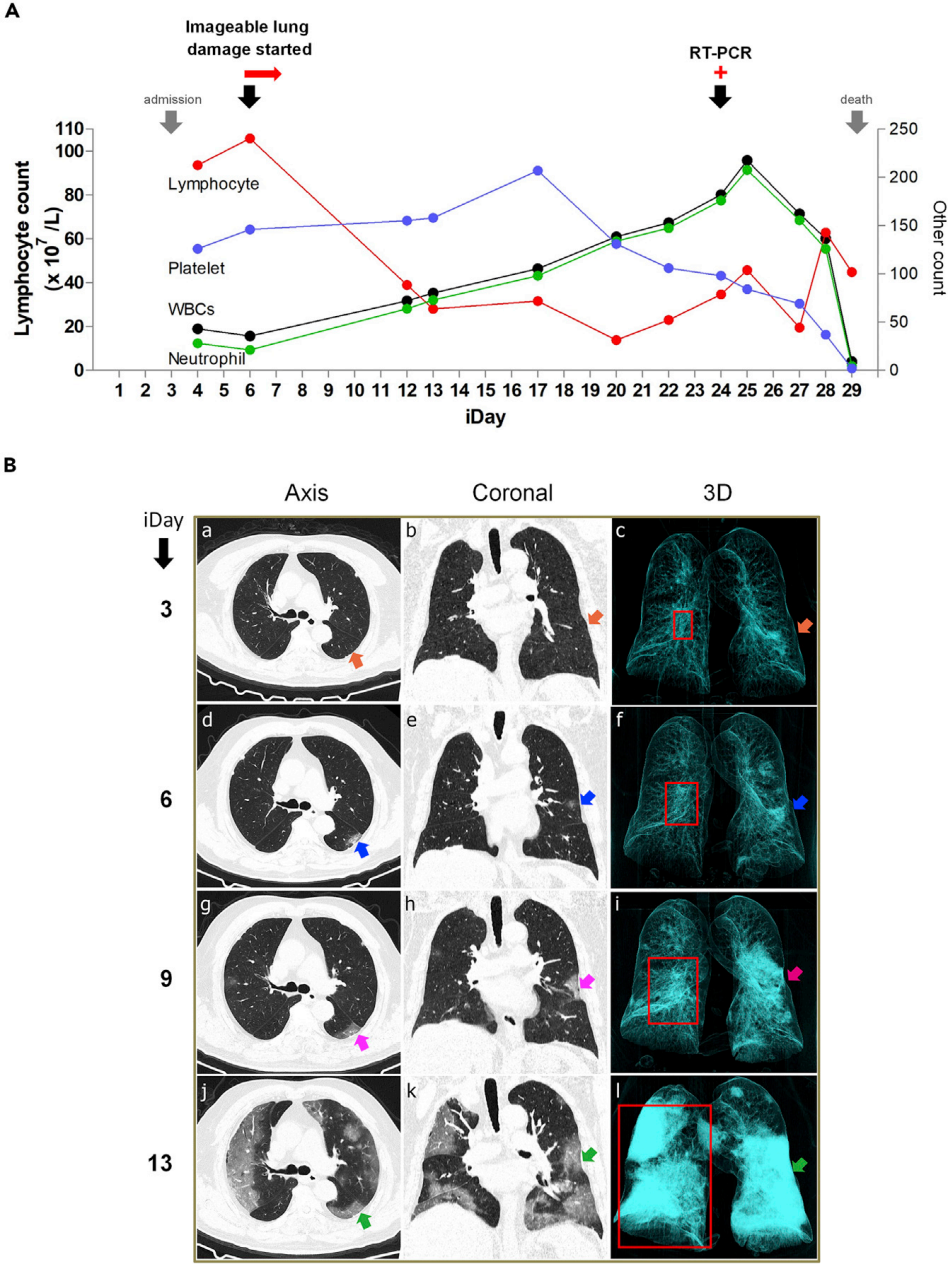
Second week crash by immune cell counts (a) and CT-based disease severity (b) in a death case (A) A different patient was admitted on iDay 3 and passed away on iDay 29. His lung damage started on iDay 6, and RT-PCR was able to detect SARS-CoV-2 RNA on iDay 24 (negative on iDays 5 and 9); cells counted were lymphocyte (red, x 10^7^/L), platelet (blue, x 10^9^/L), white blood cells (WBCs, black, x10^8^/L), and neutrophil (green, x 10^8^/L). (B) Chest CT scan imaging of his disease progression in our time points: iDays 3 (a–c), 6 (d–f), 9 (g–i), and 13 (j–l); blue arrows in d–f: new ground-glass opacities in the left lower lobe; purple arrows in g–i: original lesion enlarged; green arrows in j–l: more severe, progressive bilateral ground-glass opacities involving the subpleural area. Red boxes: disease progression in the right lung.

A 65-year-old man was chosen with no significant past medical history but fatigue for 3 days, cough, and low-grade (37.8°C) fever for 1 day. The choice of this patient was due to three parts of causes. First of all, his age accounted for the largest proportion of COVID-19 deaths, which was of important clinical significance. Second, he had no other underlying diseases, and the confounding factors and influence of other diseases on our target index could be excluded to the maximum extent. Thirdly, his changes of index were significant and representative, with complete clinical data (i.e., computed tomography [CT] images and clinical symptoms), which was more convincing. He was hospitalized on illness day 3 (iDay 3). His clinical course from iDay 4 to iDay12 was relatively stable except intermittent fevers, nonproductive cough, abdominal discomfort, and diarrhea; however, he developed progressive leukocytosis with predominant neutrophils and lymphocytopenia (Figure 4A). He had elevated CRP through the disease course. His PT was mildly elevated and went up by the last days; his AST and ALT were within normal limits until the last 2–3 days (no LDH information was available). Chest CT showed progressive lung damage from iDay 6 (Figure 4B), and his reverse transcriptase–polymerase chain reaction (RT-PCR) results came back positive on iDay 24. On iDay 28, he had multiple-organ failure and secondary bacterial infection. He had cardiopulmonary arrest and died on iDay 29.

As shown in Figure 4, the best correlations were between CT or RT-PCR findings of disease severity and the WBC subset cells’ densities, as the disease progressed. Once the lung damage was detected, lymphocyte count started dropping on the same day and never recovered. His neutrophil count represented almost all of his WBCs’ counts after iDay 13 when his lungs were progressively damaged (Figure 4B) until iDay 25. This patient had very high neutrophils but very low lymphocytes. This patient failed to clear the SARS-CoV-2 virus with persistent positive RT-PCR. The platelet count was a negative control. Various therapies available at that time were given (not listed here) without success.

### Neutrophil densities were correlated with hospitalization duration before death

**Figure 5:**
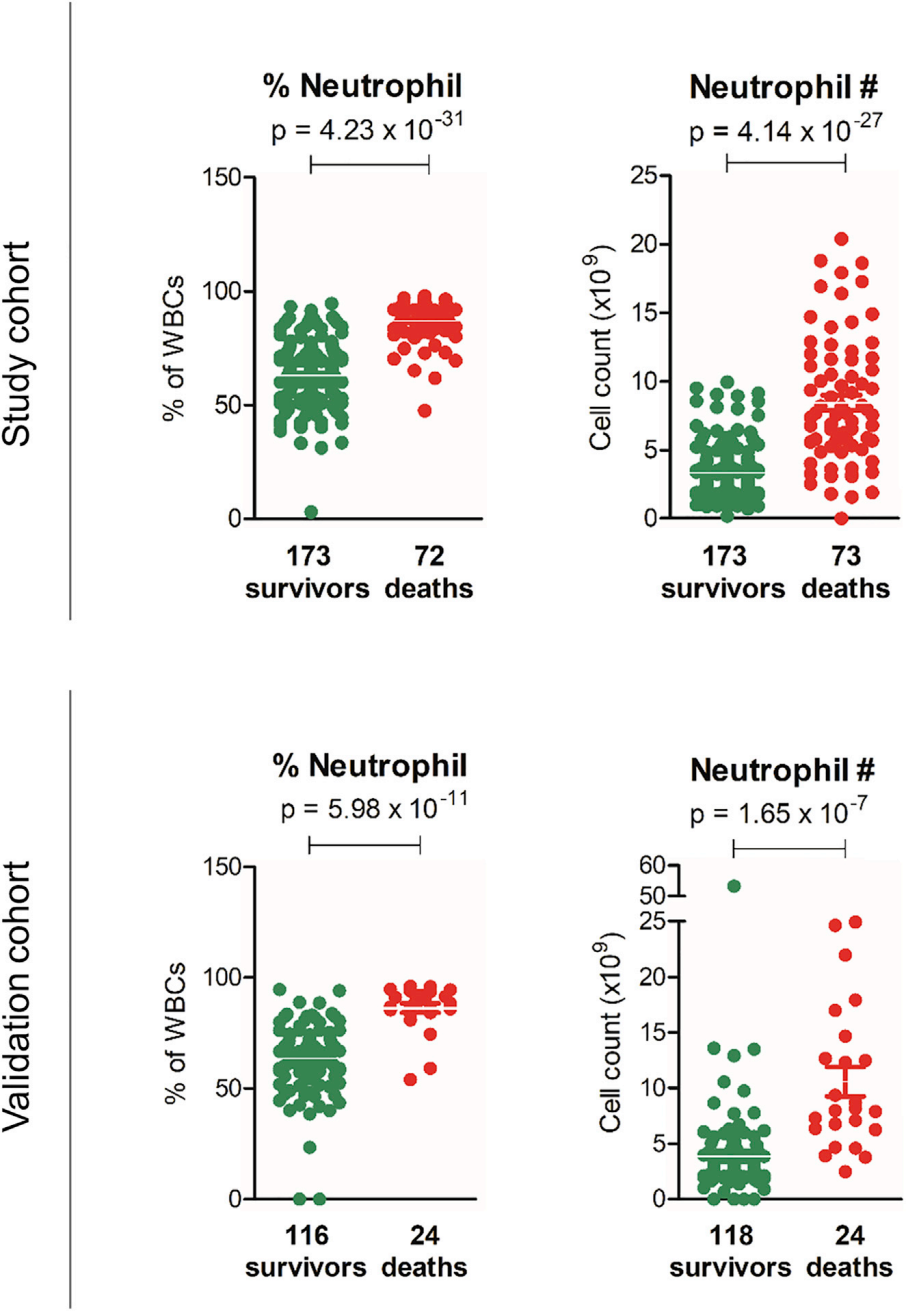
Differential distribution of two mortality-correlated neutrophil measures between surviving (green) and death (red) groups *Upper panels*, study cohort; *lower panels*, validation cohort; p values from two-tailed t tests of averages.

To study the correlational findings of WBC, neutrophil count, neutrophil percentage, and lymphocyte percentage with the mortality, group-based distributions were further evaluated to see whether the neutrophil abundance might be associated with the mortality. Based on the results in Figures 3 and 4, the top two parameters were considered: neutrophil percentage and neutrophil count. We compared the surviving group and the deceased group. Both neutrophil percentage and neutrophil count affirmed visually differential distributions between the two groups (Figure 5, *top panels*). To validate this finding, we registered another cohort of 143 patients, including 119 survivors and 24 death cases. The data were analyzed separately, and the results were shown in Figure 5 *lower panels*. Data from both cohorts were consistent with each other, confirming the dysregulated neutrophil densities were correlated with hospitalization duration before death.

## Discussion

We carried out the first *Pearson*’s correlation (quantitative) study of 27 clinical parameters to clarify prognostic markers for hospital outcomes including mortality in initial Chinese COVID-19 patients. Severe COVID-19 cases involve systemic damage and multiple-organ failure with high mortality rate. We postulated that there were some systemic parameters that we could delineate from the retrospective cohort study database. We found the dysregulated neutrophil densities were correlated with hospitalization duration before death. Surged neutrophil abundance was a poor prognostic marker for severe COVID-19 patients, supporting the findings from previous qualitative studies (reviewed by Reusch et al., 2021).

In general, the clinical parameters that serve as prognostic markers are determined by their concentration-dependent correlations with patients’ outcome. Interestingly, different sets of measures index different aspects of the prognosis, including surviving or not (indexing); if surviving, how long the recovery will take (length of hospital stays); if not, how long the patient will remain critically ill (hospitalization duration before death). The correlation directions of these parameters are then cross-verified in various settings for consistency. For example, increased neutrophil percentage is correlated with shorter hospitalization duration before death. It is also positively correlated with longer hospital stay for the surviving group (p = 0.0021). These correlational analyses thus reveal the common pathogenicity factors of COVID-19.

Surged neutrophils are constantly observed in our Chinese cohort study, likely due to their roles in pro-inflammatory response to SARS-CoV-2 infection (Perlman and Dandekar, 2005). Previous studies in Brazilians have suggested that low lymphocyte-to-neutrophil ratio predicted the mortality (Rizo-Téllez et al., 2020). Our results suggest that neutrophils may play a key role in worsening the systemic damage and multiple-organ failure in Chinese COVID-19 patients. Surged neutrophils, especially the absolute abundance rather than its percentage, are correlated with both hospitalization duration and mortality in the deceased group; this is consistent with the findings that accumulation of cytotoxic substances from neutrophil’s lethal cargo may cause massive destruction to host tissue (Amulic et al., 2012). As shown in our “second week crash” deceased case, SARS-CoV-2 virus dysregulated the immune system by increasing the neutrophil concentration, at the same time decreasing the abundance of lymphocyte. Such dysregulation could cause triple threats: (1) suppressing the antiviral activity of lymphocyte, (2) excessive production of cytotoxic substances, and (3) neutrophil’s potential negative effect on remaining lymphocyte (Costa et al., 2019; Pillay et al., 2012). The triple threats may help explain the poor prognosis for the severe COVID-19 patients. Interestingly, as opposing to the literature (Liu et al., 2020b), NLR did not show a significant role in those cohorts, suggesting again that the abundance of neutrophils, not the ratio (Liang et al., 2020), contributed to the poor prognosis.

Acute respiratory distress syndrome (ARDS) is a common complication of SARS-CoV-2 infection. It is not only the lung epithelial damage caused by viral infection but also the patients’ immune responses that contribute to disease severity. Patients with severe COVID-19 often have cytokine storm, with massive inflammatory damage leading to multi-organ failure. Pro-inflammatory signatures of severely affected COVID-19 patients feature elevations of interleukin-1β (IL-1β), IL-6, and tumor necrosis factor alpha (TNF-α) (Liu et al., 2020a). Early studies showed the degree of increase of neutrophil percentage in the blood correlates with the severity of COVID-19 (Del Valle et al., 2020). There are several clinical trials targeting neutrophils to treat severe COVID-19 with ARDS (Chiang et al., 2020).

Many clinical trials are targeting neutrophils among other related cell type in COVID-19. As of middle September 2021, more than 10,000 clinical trials on COVID-19 were downloaded from the three major registries, and inter-registry duplicates were removed. As a result, 4,000 of them were interventional in search for effective treatments, including 2,000 from clinicaltrials.gov, 300 from EU Clinical Trials Register, and 1,000 from the WHO registry. Two hundred and one (5%) of them were designated to use drugs, including 19 that could target 15 different molecules expressed by neutrophils in more than 100,000 patients, located in more than 46 countries (Table 2). Those drugs carried at least six mechanisms, including attenuating neutrophil infiltration (ifenprodil, in two clinical trials), reducing neutrophil abundance (secukinumab, in two trials), blocking neutrophil activation (eight drugs including ruxolitinib and baricitinib in 63 trials), blocking chemokine and other chemoattractant functions (two drugs in five trials), other inhibition mechanisms (six drugs including tocilizumab in 134 trials), and degrading extracellular traps (rhDNase I, one trial). Algernon Pharmaceuticals Inc of Canada got US Food and Drug Administration (FDA) approval in June of 2020 for repurposing ifenprodil, an N-methyl-D-aspartate (NMDA) receptor antagonist, to treat COVID-19 in multinational phase 2/3 trials with 168 patients, obtaining positive results indeed. The repurposing idea was partly to reduce the infiltration of neutrophils and T cells into the lungs where they could release glutamate and cytokines, respectively. The latter might result in cytokine storm, a critical characteristic of severe COVID-19 (Kim et al., 2021). Tocilizumab, targeting the IL-6 receptor, is the major treatment of interest, as 122 trials have been evaluating it to treat COVID-19.

**Table 2:** Clinical trials targeting neutrophil in patients with COVID-19^a^

Mechanism	Target	Drug^b^	Country^c^	Phase	Enrollment	Status	Trial ID^d^
Attenuating infiltration	NMDA receptors	Ifenprodil	Four	2, 3	168	Completed	**NCT04382924**
			Korea	2	40	Ongoing	KCT0005307
Reducing neutrophil density	IL-17a	Secukinumab	Spain	4	800	Ongoing	2020-001,357-52
			Russia	2	70	Ongoing	NCT04403243
Blocking neutrophil activation	FPR1	Cyclosporin H	USA	2	75	Ongoing	NCT04492891
			USA	1	20	Completed	NCT04412785
	Tyrosine kinase SYK	Fostamatinib	Multisite	3	308	Ongoing	NCT04629703
			Great Britain	2, 3	186	Completed	2020-001750-22
			USA	2	59	Completed	*NCT04579393*
			UK	1, 2	456	Ongoing	NCT04581954
	JAKs	Ruxolitinib^e^	UK	4	375	Ongoing	ISRCTN11188345
			USA	3	432	Completed	*NCT04362137*
			Multisite	3	402	Completed	2020-001662-11
			France	3	54	Ongoing	2020-001963-10
			France	3	216	Not yet recruiting	NCT04424056
			Multisite	3	60	Not recruiting	PER-030-20
			Great Britain	2, 3	186	Completed	2020-001750-22
			Germany	2, 3	200	Completed	2020-001481-11
		Baricitinib^f^	Great Britain	4	59	Completed	2020-001777-71
			USA	3	1033	Completed	**NCT04401579**
			Italy	3	12	Completed	NCT04358614
			Italy	?	20	Completed	NCT04438629
			Multisite	3	1525	Completed	**NCT04421027**
			Multisite	3	1010	Completed	NCT04640168
			Bangladesh	3	150	Ongoing	NCT04693026
			UK^g^	3	50000	Ongoing	NCT04381936
			Italy	2, 3	200	Unknown	NCT04320277
		Tofacitinib	Spain	4	800	Ongoing	2020-001357-52
			Italy	2	116	Ongoing	2020-002035-30
			Six	2	40	Ongoing	2018-000930-37
			Brazil	2	289	Completed	NCT04469114
			USA	2	24	Terminated	NCT04415151
			Italy	2	116	Not yet recruiting	NCT04390061
			Italy	2	50	Unknown	NCT04332042
	GM-CSF	GSK3196165	Three	2	782	Completed	**2020-001759-42**
			Seven	2	1156	Completed	**NCT04376684**
	GM-CSF receptors	CAM-3001	Seven	2,3	115	Ongoing	PER-032-20
			Four	2,3	815	Completed	NCT04447469
			USA	2	60	Ongoing	NCT04492514
			USA	2	2	Completed	NCT04463004
			Italy	2	50	Not yet recruiting	NCT04397497
			USA	2	40	Completed	*NCT04399980*
	BTK inhibitor	Ibrutinib	USA	2	46	Completed	NCT04375397
			USA	2	10	Ongoing	NCT04439006
			USA	2	0	Withdrawn	NCT04665115
Blocking chemokine and other chemoattractant functions	C5a	Eculizumab	France	2	120	Unknown	NCT04346797
			USA	?	?	No longer available	NCT04355494
			USA	?	?	Available	NCT04288713
	CXCRs	Reparixin	Italy	3	303	Ongoing	2020-005919-51
Other inhibition	IL-6 receptor	Tocilizumab^h^	45	0–4	>79467	Various	122 clinical trials
	β1-Adrenergic receptor	Metoprolol	Spain	2	20	Ongoing	2020-002310-41
			USA	?	22213	Completed	NCT04467931
	TNF	Adalimumab	Spain	4	800	Ongoing	2020-001357-52
			?	3	0	Withdrawn	NCT04705844
			Great Britain	2	750	Completed	2020-003628-18
			Great Britain	2	1500	Completed	2020-004144-28
			Six	2	40	Ongoing	2018-000930-37
		Golimumab	Spain	4	800	Ongoing	2020-001357-52
			Six	2	40	Ongoing	2018-000930-37
		Infliximab	Spain	4	800	Ongoing	2020-001357-52
			Great Britain	3	168	Completed	2020-001684-89
			USA	3	2160	Ongoing	NCT04593940
			Great Britain	2	1500	Completed	2020-004144-28
			Six	2	40	Ongoing	2018-000930-37
			USA	2	17	Completed	NCT04425538
			France	?	850	Ongoing	NCT04344249
	Elastase	Alvelestat	USA	1, 2	15	Completed	NCT04539795
Degrading extracellular traps	Extracellular traps	rhDNase I	Canada	1	25	Ongoing	NCT04409925

a ?, information unavailable.

b not necessarily neutrophil-selective.

c number indicates multiple countries involved.

d not listed are 12 phase 2 trials.

e not listed are seven phase 2 trials.

f one of several drugs in 40,000 patients.

g details not listed here.

h bold: statistically significant efficacy p < 0.05; italic: insignificant p > 0.05, as per posted results.

Only few other studies have obtained early results, especially for the popular drug tocilizumab. Tocilizumab (8 mg/kg i.v. for 4 weeks) alone was able to slow down the progression of the disease, compared with placebo in a 389 US minority patients trial, which represents the largest cohort so far (Salama et al., 2021), but not in Brazilian, Italian, or French patients of much smaller cohorts (Hermine et al., 2021; Salvarani et al., 2021; Veiga et al., 2021). Preliminary results from two other Italian trials and a Chinese trial all favors the use of this drug, based on the observations of reduced IL-6 levels, increased PaO2/FiO2 values, improving pulmonary inflammation, inhibiting disease progression, and lowering lethality rate (Perrone et al., 2020; Pomponio et al., 2021; Zhao et al., 2021). Furthermore, ruxolitinib that targets JAKs has been shown to be effective for treating tocilizumab-refractory COVID-19 (Innes et al., 2020) as well as Chinese patients (Cao et al., 2020). Another JAK inhibitor, baricitinib, has also been shown to be able to help and prevent this disease from progression to severe forms and reduce mortality, partly by restoring normal-abundance neutrophils (Bronte et al., 2020; Kalil et al., 2020; Marconi et al., 2021) (Table 2), and consistently, tofacitinib showed similar therapeutic effects (Guimarães et al., 2021). None of the other drugs have any treatment results available yet. The very early results from three drugs binding to IL-6 and JAKs thus already prove the efficacy of targeting neutrophils and T cells in treating COVID-19. Of the note, two studies targeting GM-CSF both obtained positive results with GSK3196165 (Table 2). From other clinical trials, more positive results are anticipated, as for fostamatinib that blocks neutrophil activation (Strich et al., 2021). Such information suggests that our findings in Chinese patients may apply to other ethnicities, which warrants additional Pearson’s analysis.

### Limitations of the study

This study has several limitations such as lack of ethnicity comparison. Stages of the disease at admission and comorbidity were not included in the correlations, and correlations alone do not demonstrate causality. Longitudinal study involved N of 1. Cellular morphology, low versus normal abundance subpopulation (neutrophil subsets), was not examined. In general, the mechanism for the neutrophil surges was not explored for this retrospective study. Furthermore, unlike an interventional clinical trial, this retrospective study by design was set to test a correlation between two continuous variables. Therefore, such correlation did not demonstrate a causal effect.

## STAR★Methods

### Key resources table

**Table:** 

REAGENT or RESOURCE	SOURCE	IDENTIFIER
**Biological samples**		

oropharyngeal swabs	WRCH	N/A
blood	WRCH	N/A

**Critical commercial assays**		

multigene-based 2019-nCoV Nucleic Acid Detection Kit	DAAN Gene Co., Ltd.	N/A

**Oligonucleotides**		

CoV-N-P	5′-FAM-TTGCCCCCAGCGCTTCA-BHQ1-3′	N/A
CoV-N-F	5′-TTGGGGACCAGGAACTAAT-3′	N/A
CoV-N-R	5′-GAAGGTGTGACTTCCATGC-3′	N/A
ORF1ab-P	5′ HEX-TCCCACCCAAGAATAGCATAGATGC-BHQ1-3′	N/A
ORF1ab-F1	5′-TTTAGATATATGAATTCACAGGGA-3′	N/A
ORF1a-R1	5′-ACCAACACCCAACAATTTAAT-3′	N/A
RNP-P	5′Cy5-TCCACAAGTCCGCGCAGAG-BHQ2-3′	N/A
RNP-F	5′-AGATTTGGACCTGCGAG-3′	N/A
RNP-R	5′-ACTGAATAGCCAAGGTGAG-3′	N/A

**Software and algorithms**		

SPSS	IBM	http://www.spss.com/
Graphpad Prism5	Graphpad	https://www.graphpad.com/

### Resource availability

#### Lead contact

Further information and requests for resources and reagents should be directed to and will be fulfilled by the lead contact, Zhicheng Lin (zhicheng_lin@hms.harvard.edu).

#### Materials availability

This study did not generate new unique reagents.

### Experimental model and subject details

#### Human subjects and study setting

This retrospective, noninterventional study consisted of COVID-19 patients who were admitted to WRCH during the initial outbreak of COVID-19 from January to March of 2020 in Wuhan of China. No selection of subjects was made based on age or gender. The study was performed in accordance with the Declaration of Helsinki and national and institutional standards. The study was approved by the Institutional Review Board, namely, the WRCH Ethics Committee.

All patients were tested positive at the time of admission, according to the fifth version guidelines of COVID-19 diagnosis and treatment in China. Study cohort and validation cohort were randomized. The study group consisted of 74 patients who died from COVID-19 and 174 patients who survived COVID-19. All patients were confirmed with COVID-19 by positive RT-PCR for SARS-CoV-2. Outcomes are the length of hospital stay before discharge or before death. The study protocol was approved by the WRCH Ethics Committee. Written informed consent was obtained from study participants for the publication of any potentially identifiable images or data included in this article.

### Method details

#### CT scan

As reported before (Li et al., 2020), each chest was scanned in 1-mm slice thickness CT on a Siemens SOMATOM go.Top 64 scanner (Siemens Healthineers, Suzhou, China), by using a field of view (FOV) 41.3 × 41.3 cm, tube voltage 130 kV and current 138 mA, pitch 0.6, reconstruction layer thickness 1.5 mm.

#### RT-PCR

Oropharyngeal swabs were collected multiple times following hospital admission and PCRed for the presence of SARS-CoV-2 by using two kits from DAAN Gene Co., Ltd. of China. Oropharyngeal swabs were each collected into a tube with 200 μL of virus preservation solution, and total RNA was extracted within 2 h. After standing at room temperature for 30 min, the swab was spun down at 8,000 rpm. The suspension was used for RT-PCR assay in a multigene-based 2019-nCoV Nucleic Acid Detection Kit. Three target genes, including nucleocapsid protein (N), open reading frame 1ab (ORF1ab) and ribonucleotide-protein (RNP), were amplified and tested simultaneously during the PCR assay by using the following primers:

for N-gene:

probe was CoV-N-P: 5′-FAM-TTGCCCCCAGCGCTTCA-BHQ1-3′
Forward primer CoV-N-F: 5′-TTGGGGACCAGGAACTAAT-3′
Reverse primer CoV-N-R: 5′-GAAGGTGTGACTTCCATGC-3′

for ORF1ab-gene:

probe was ORF1ab-P: 5′ HEX- TCCCACCCAAGAATAGCATAGATGC-BHQ1-3′
forward primer ORF1ab-F1: 5′-TTTAGATATATGAATTCACAGGGA-3′
reverse primer ORF1a-R1: 5′-ACCAACACCCAACAATTTAAT-3′

for RNP-gene:

probe was RNP-P: 5′Cy5- TCCACAAGTCCGCGCAGAG-BHQ2-3′
forward primer RNP-F: 5′-AGATTTGGACCTGCGAG-3′
reverse primer RNP-R: 5′-ACTGAATAGCCAAGGTGAG-3′.

PCR assay was performed under the following conditions: 15 min incubation each at 50°C and 95°C, 45 cycles of 15 sec denaturation at 94°C, and extending and collecting fluorescence signal at 55°C for 45 s. Cycle threshold values were collected and a cycle threshold value (Ct-value) less than 40 was defined as a positive. A positive result was validated at two institutions: Union Hospital, Tongji Medical University, Huazhong university of Science and Technology and the ADICON clinical laboratories.

#### Clinical management

All patients were hospitalized at WRCH and received standardized care according to the guideline at that time (Diagnosis and treatment plan of Corona Virus Disease 2019 (tentative sixth edition), 2020). At admission, complete blood cell count, blood chemistry panels, renal and liver functions, and C-reactive protein were tested. Respiratory samples were tested for influenza and other respiratory viruses with a multiplex PCR assay. During hospital stay, all patients received standardized care for COVID-19 patients, including supplemental oxygen therapy, non-invasive mechanical ventilation and invasive mechanical ventilation for acute respiratory failure. Some of them received short course (3–5 days) of glucocorticoids (1 mg/kg per day) as per guideline. Some of them received antibiotics if they have persistent fever for more than 3–5 days. Some of them received traditional Chinese medicine treatment.

#### Data retrieving

A trained team of physicians reviewed and collected clinical and outcomes data from electronic health records at WRCH. All the individual components of the database were recorded and checked separately by two independent physicians. Data were summarized using a standardized database collection form. Information retrieved from the collected data was reviewed and cross-checked for accuracy by two additional trained physicians (N.X. and J.L.). No data were excluded from this study and all de-identified data in associated table and figures are available.

#### Search of clinical trials databases

Terms “COVID-19”, “neutrophil”, “drug”, “treatment” and “intervention” were used to search three clinical trial databases, the US database (clinicaltrials.gov), the European database (clinicaltrialsregister.eu) and the WHO International Clinical Trials Registry (who.int/trialsearch) for treatments of COVID-19. Trials related to drugs targeting neutrophils, included those previously reviewed (Németh et al., 2020), were retrieved and summarized.

### Quantification and statistical analysis

Categorical variables were described as frequency rates and percentages. Data were expressed in mean ± s.e.m. (standard error of the mean), as shown in Table 1. SPSS (Statistical Package for the Social Sciences, version 25) or algorithms implemented in Prism GraphPad (v5 or v8) were used for data analyses, including linear and nonlinear (plateau followed by one phase decay) modeling of correlations between measures and hospital stay, and estimation of average Pearson correlation coefficient (*r*) for fitting of the correlation with 95% confidence interval (95%CI), as shown in Figures 2, 3 and S1. Covariates were tested systematically via multiple linear regressions and F-tests. Statistical analyses used *Student*’s two-tailed t-tests (Figure 5), χ^2^ tests or Fisher’s exact tests (Table 1). p < 0.05 was considered as statistically significant, with Bonferroni for multiple-testing in Figures 2, 3 and S1.

## Data Availability

Anonymous human participant research data used in this report was designated for this study, and further data sharing is restricted and not publicly available. No formatted data types are detailed in this manuscript. Data requests could be submitted to the lead contact, evaluated by and require an agreement of the Ethics Committee. Summary statistics are already shown in the main Manuscript. Any additional information required to reanalyze the data reported in this paper is available from the lead contact upon request.
